# Stigmasterol: An Enigmatic Plant Stress Sterol with Versatile Functions

**DOI:** 10.3390/ijms25158122

**Published:** 2024-07-25

**Authors:** Julia Valitova, Albina Renkova, Richard Beckett, Farida Minibayeva

**Affiliations:** 1 Kazan Institute of Biochemistry and Biophysics, FRC Kazan Scientific Center of RAS, P.O. Box 261, Kazan 420111, Russia; yulavalitova@mail.ru (J.V.); renkova@kibb.knc.ru (A.R.); 2 School of Life Sciences, University of KwaZulu-Natal, Private Bag X01, Scottsville 3209, South Africa; rpbeckett@gmail.com

**Keywords:** stigmasterol, C22-sterol desaturase, plants, lichens, stress

## Abstract

Sterols play important structural and regulatory roles in numerous intracellular processes. Unlike animals, plants contain a distinctive and diverse variety of sterols. Recently, information has emerged showing that stigmasterol is a “stress sterol”. Stigmasterol is synthesized via the mevalonate biosynthesis pathway and has structural similarity to *β*-sitosterol but differs in the presence of a trans-oriented double bond in the side chain. In plants, the accumulation of stigmasterol has been observed in response to various stresses. However, the precise ways that stigmasterol is involved in the stress responses of plants remain unclear. This comprehensive review provides an update on the biology of stigmasterol, particularly the physicochemical properties of this ethylsterol, its biosynthesis, and its occurrence in higher plants and extremophilic organisms, e.g., mosses and lichens. Special emphasis is given to the evolutionary aspects of stigmasterol biosynthesis, particularly the variations in the gene structure of C22-sterol desaturase, which catalyzes the formation of stigmasterol from *β*-sitosterol, in a diversity of evolutionarily distant organisms. The roles of stigmasterol in the tolerance of plants to hostile environments and the prospects for its biomedical applications are also discussed. Taken together, the available data suggest that stigmasterol plays important roles in plant metabolism, although in some aspects, it remains an enigmatic compound.

## 1. Introduction

Plants, unlike animals, produce a complex array of sterols. This diversity is probably a consequence of the various roles that they play in plant life [1,2]. All fungal and mammalian cells contain one major sterol, ergosterol or cholesterol, respectively, while in contrast, plants synthesize numerous sterols, with *β*-sitosterol, stigmasterol, and campesterol being most important. It is unclear why plants, unlike other organisms, require a mixture of sterols [3]. In plants, sterols are present as free sterols, as sterols coupled with higher fatty acids (sterol esters), and as steryl glycosides and acylated steryl glycosides, the carbohydrate derivatives of sterols. Such conjugates are rarely found in animal cells. The relative content of the various conjugated sterols differs from species to species and may vary under different environmental conditions [4,5]. Currently, more than 200 types of plant sterols and their derivatives are known. A distinct structural feature of plant sterols is the presence of methyl or ethyl groups at C24 of the side chain. On this basis, plant sterols are divided into 24-methyl- and 24-ethylsterols, where *β*-sitosterol and stigmasterol are ethylsterols, while campesterol is a methylsterol. Changes in the ratio of 24-methyl- to 24-ethylsterols occur as part of the response of plants to stress factors. It is now becoming clear that, along with their structural functions, sterols play important regulatory roles in various intracellular processes. Some sterols, especially *β*-sitosterol, display antioxidant properties. Sterols are also the main components of lipid rafts, which are membrane microdomains involved in cell signal transduction. Thus, sterols play important diverse functions in plants [6].

**Figure 1:**
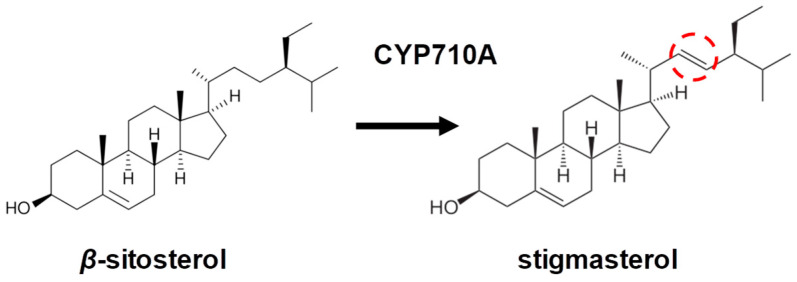
Schematic diagram of the desaturation reaction catalyzed by C22-sterol desaturase (CYP710A). Dotted oval indicates the structural difference.

Stigmasterol is an unsaturated sterol that is the end product of the phytosterol biosynthesis pathway. The precise roles of stigmasterol in plants remain unclear. However, a recent review [7] suggested that stigmasterol may play general roles in stress tolerance, based on observed changes in the stigmasterol content following pathogen infection [8], wounding [9], and during gravitropic stimulation [10]. Biosynthetically, stigmasterol is produced by the desaturation of *β*-sitosterol (Figure 1). Plants can reduce the levels of free stigmasterol by further metabolizing it to sterol conjugates such as sterol esters, sterol glucosides, and acyl sterol glucosides. The ratio of stigmasterol to *β*-sitosterol affects membrane fluidity and ion permeability and furthermore affects the activity of membrane-bound proteins including those involved in signaling [11]. The aim of the present review is to summarize the recent discoveries on the physicochemical properties of stigmasterol, its biosynthesis, and its potential roles in stress tolerance. In particular, we review the evolutionary aspects of stigmasterol biosynthesis in plants, including the enzyme C22-sterol desaturase (CYP710A), which catalyzes the final step of stigmasterol biosynthesis.

## 2. Properties of Stigmasterol as a Component of Plant Membranes

Plant sterols are important structural components of membranes and can determine their mechanical properties. Changes in sterol content and composition can influence membrane permeability, hydrocarbon chain order, condensation efficiency, and elasticity [12]. Studies on the effects of sterol composition on the properties of membranes are often carried out using synthetic membranes, which usually comprise a mixture of phospholipids and a variety of sterols [13]. The biophysical properties of these artificial membranes have been studied using a variety of approaches such as electron paramagnetic resonance (EPR), fluorescence spectroscopy, and nuclear magnetic resonance (NMR) spectroscopy [14,15]. In addition, small-angle X-ray scattering (SAXS) can be used to detect changes in bilayer thickness, effective packing area per lipid, and any “waves” that occur when lipid bilayers form in the presence of different sterols [14]. The effects of phytosterols on the properties of model membranes have been extensively studied over the years, and their effects are usually compared with those of cholesterol [13]. For example, each sterol can affect the permeability of the lipid layer of the plasmalemma to water in different ways due to their differences in chemical structure. As discussed above, stigmasterol is structurally similar to *β*-sitosterol but differs in the presence of a trans-oriented double bond at the C22 position in its side chain, making it more unsaturated. This bond reduces its solubility in the lipid bilayer as the mismatch between the trans-double bond of the sterol molecule (length: 17 A˚) and the cis-double bond of the lipid hydrocarbon chain results in stereo-chemical packing incompatibility [16]. The plant sterols *β*-sitosterol and stigmasterol can readily partition into those membranes that possess a high proportion of unsaturated lipid acyl chains. SAXS studies with cholesterol, *β*-sitosterol, and stigmasterol revealed that cholesterol had the greatest effect on membrane structural parameters such as bilayer thickness and fluctuations, followed by *β*-sitosterol and stigmasterol. The smaller effects of the latter phytosterols on the membrane state can be explained by the presence of an additional ethyl group at C24 in the side chain, which makes them less flexible when interacting with phospholipids. However, it seems likely that the presence of an additional ethyl group can enhance van der Waals interactions, leading to a higher membrane density and other changes in properties, e.g., lower temperature sensitivity. According to Dufourc [17], *β*-sitosterol and stigmasterol can be thought of as “membrane dynamics regulators” that maintain the membrane in an optimal state to enable cells to function at unfavorable temperatures. However, the presence of a double bond in the side chain (C22–23) of the stigmasterol molecule makes it more flexible compared to other plant sterols with saturated side chains, such as *β*-sitosterol. The rotational rigidity caused by the saturation of this double bond in cholesterol, campesterol, and *β*-sitosterol leads to greater order, while stigmasterol reduces order [18]. Thus, the saturation of the plant sterol side chain determines the effects that a sterol will have on the biophysical properties of lipid bilayers [19]. Based on computation modeling simulations, it seems likely that sterols with this double bond will have a thinner bilayer and looser lipid packaging, i.e., generally increased membrane fluidity [19]. However, if the common sphingolipid glycosylinositol phosphoceramide is present, it can interact with stigmasterol and increase membrane order. Conversely, if glucosylceramide is present, the resulting interaction with stigmasterol can decrease order [18]. According to Wagatsuma [20], this structural feature of stigmasterol increases the permeability of membranes to water and ions and therefore, in most cases, the greater the content of stigmasterol, the higher the membrane permeability will be.

Therefore, the changes in the content of stigmasterol can alter membrane characteristics either directly or indirectly through interactions with other membrane lipids, which can in turn effect a variety of membrane-associated metabolic processes and signaling pathways in plants during stresses.

## 3. Stigmasterol in Different Organisms

Stigmasterol is produced by almost all plants, but its relative abundance may vary between species. There are scattered reports in the literature on the total stigmasterol content of various plants. For example, in leaves of the Asiatic plant *Centella asiatica*, the stigmasterol content reaches 0.023 ± 0.70 µg mg^−1^ dry mass [21]. Chowdhary et al. [22] reported that the plant *C. asiatica* collected from Panvel, India, was the best source of stigmasterol (0.0582%) compared to plants collected from other different regions of India. Sethiya and Mishra [23] reported that the levels of stigmasterol in *Evolvulus alsinoides*, *Convolvulus pluricaulis*, *Clitoria ternatea*, and *Canscora decussata* were much higher, at 93, 155, 32, and 39 µg g^−1^ dry mass, respectively.

In mosses, interestingly, the proportion of stigmasterol in membrane lipids is typically greater than that in higher vascular plants; it is often even the predominant sterol. Comparisons of the genomes of model plant systems have provided key evidence of the evolution and diversity of genes involved in the sterol biosynthesis pathways, and may help explain why such a diversity of plant sterols has been preserved during the evolution of terrestrial plants. In particular, the moss *Physcomitrium (Physcomitrella) patens* has been used as a model to elucidate the pathways of sterol biosynthesis in evolutionarily ancient plants. Bryophytes occupy a phylogenetic position between green algae and vascular plants. Their ancestors are believed to have diverged shortly after the transition from water to land, at least 500 million years ago (Mya) [24]. Although bryophytes are minor components of most terrestrial ecosystems, they dominate in areas with unfavorable climatic conditions, for example, in arid steppes, arctic, subarctic latitudes, and in the Antarctic. To survive in harsh conditions, mosses have developed effective mechanisms to tolerate dehydration, high temperatures, and low nutrient supplies. As a result, mosses are often referred to as extremophile plants (from the Latin extremus meaning “extreme”, and the Greek philiā meaning “love”). Extremophiles are therefore organisms that can survive in extreme conditions, conditions that would be highly unfavorable for other organisms. It is interesting that the composition of sterols in mosses differs significantly from that of higher vascular plants. Interestingly, whereas, as discussed above, stigmasterol is a minor sterol in unstressed higher plants, it is a major sterol in *P. patens*, suggesting taxonomical differences in the stigmasterol/*β*-sitosterol ratio that may have adaptive significance [25]. In *P. patens*, stigmasterol may comprise as much as 56–60% of the total amount of sterol content with *β*-sitosterol comprising 8–12%. In contrast, in higher plants, stigmasterol comprises only a small proportion of the total sterol content [25]. It is tempting to suggest that the high content of stigmasterol in bryophytes is involved in their high stress tolerance, although the exact biological significance of *β*-sitosterol C22 desaturation in plants remains unknown. 

Interestingly, stigmasterol is found not only in plants, but in another organisms. For example, while in many lichens, what are also extremophile organisms, stigmasterol is rare; in some species, several forms of stigmasterol are found, e.g., in *Lobaria pulmonaria*, *Usnea articulata*, *Cladonia furcata*, *Collema* cf. *policarpon*, and *Umbilicaria cylindrica* [26]. However, in general phytosterols have rarely been reported from fungi, although there are some exceptions, e.g., [27,28,29]. Carvalho et al. [30] also reported the isolation of the phytosterols *β*-sitosterol, stigmasterol, and sitostenone from the endophytic fungus *Colletotrichum gloeosporioides*.

Algae have a diverse sterol composition. For example, red algae (Rhodophyta) contain primarily cholesterol, but several species contain desmosterol and 22-dehydrocholesterol. Fucosterol is the dominant sterol of brown algae (*Phaeophyta*), while green algae (*Chlorophyta*) contain chondrillasterol, poriferasterol, 28-isofucosterol, ergosterol, cholesterol, and others [31,32]. The factors determining sterol differences between algal species could be morphological, environmental, or evolutionary. Stigmasterol is rarely found in the sterol pool of algae, but the presence of this sterol has been shown in the highly stress-tolerant unicellular alga *Coccomyxa subellipsoidea*, which can grow at extreme, near-polar temperatures [33]. Thus, although stigmasterol is a specific sterol of higher plants, it can also be found in other organisms, including extremophilic organisms, such as mosses, lichens, and some algae.

## 4. C22-Sterol Desaturase (CYP710A) as a Key Enzyme of Stigmasterol Biosynthesis

The sterol biosynthetic pathway in plants is a complex and multistep process starting from the initial stage of the conversion of acetate to squalene, the common precursor of all sterols and triterpenoids, via the mevalonic acid (MVA) pathway. This is followed by the cyclization of the oxidized linear precursor (2,3-oxosqualene) to cycloartenol and its further conversion to various end products. Stigmasterol one of the end products of the mevalonate pathway. The main enzymes involved in post-oxidosqualene cyclization of plant sterols are C24-sterol methyltransferase (SMT) and CYP710A. The phytosterol side chain usually has an additional methyl or ethyl group at the C24 position. There are two different isoforms of SMT enzymes (SMT1 and SMT2); these isoforms are localized in the endoplasmic reticulum and are involved in primary and secondary C24 methylation. SMT2 activity determines the ratio of 24-methyl- and 24-ethylsterols, and this ratio is believed to regulate plant ontogeny and the response of plants to stress factors [6]. Secondary methylation is the last step in sterol biosynthesis and leads to the formation of 24-ethylsterols such as *β*-sitosterol and stigmasterol [34,35]. The conversion of *β*-sitosterol to stigmasterol is catalyzed by CYP710A through the formation of a double bond at C22 [5,6,36]. It has been shown that the activity of CYP710A increases under the action of various stress factors, for example, during an attack by a pathogen [8,37].

Stigmasterol levels in plants can be increased by mutating the hydra/fackel genes that encode sterol isomerase Δ8–Δ7 and sterol C-14 reductase, respectively. hydra/fackel mutants of *Arabidopsis* with elevated stigmasterol levels display disrupted cellular processes during plant growth and development, which lead to defective embryonic morphogenesis [38,39,40]. In addition, *Arabidopsis* hydra 2/fackel and acbp1 mutant plants with increased stigmasterol levels also display the “bare root” phenotype. This may be the result of the ectopic expression of GL2 (GLABRA2), a phospholipid/sterol-binding transcription factor, which is normally involved in the regulation of root hair development in trichoblasts [39,41,42].

Conversely, the absence of stigmasterol does not necessarily result in growth or developmental defects. For example, recent work by Aboobucker et al. [43] demonstrated that maize with mutated Zmcyp710a8, which is unable to produce stigmasterol, grows normally and produces seeds. Similarly, the tomato stigmasterol Lecyp710a11 [44] and *Arabidopsis* Atcyp710a1 [35,45] mutants grow well and produce seeds under controlled conditions. Thus, in unstressed plants, stigmasterol plays a minor and potentially “vestigial” function in both monocot and dicot species.

The CYP710 family is one of four P450 families that also comprises CYP51, CYP97, and CYP711. This enzyme occurs in algae to higher plants, suggesting that C22 desaturation is probably important for all plant taxa [46]. As discussed above, the precise role of plant C22-sterol desaturase or CYP710A is to catalyze the desaturation of the side chain of *β*-sitosterol moleculed at C22, resulting in the formation of stigmasterol. CYP710A proteins have strong substrate specificity for 24-alkyl groups [35]. Membrane status can be controlled by changing the ratio of *β*-sitosterol to stigmasterol [45,47]. In the *Brassicaceae*, including *Arabidopsis*, the substrates for CYP710A are 24-methylsterols (campesterol/24-epi-campesterol) and 24-ethylsterols (*β*-sitosterol). The resulting products are the corresponding ∆22-sterols, stigmasterol, and brassicasterol/crinosterol, respectively.

The number of genes encoding C22-sterol desaturase varies in different plant species; for example, in *Arabidopsis*, there are four genes belonging to the CYP710A family [1,35]. Multiple copies of sterol C22 desaturase genes have been found in mosses [25] and poplar [48]. In contrast, maize has a single sterol C22 desaturase [43]. In the hexaploid wheat Triticum aestivum, the C22-sterol desaturase genes are present as three homoeologous copies [7]. Recently, we showed that homoeologous genes encoding C22-sterol desaturase (CYP710A8) display different patterns of co-expression in the roots and leaves of wheat seedlings following exposure to abiotic stresses [49,50].

In fungi, the CYP61 family is equivalent (orthologous) to the CYP710 family in plants [51]. CYP710 and CYP61 have recently been recognized as belonging to a single clan, and it has even been suggested that these two CYP families should be grouped together [52]. The separation of these families pre-dated the first split that led to plant–fungal divergence, and probably occurred at around 1100 Mya based on the evolutionary distance between CYP61 and CYP710. Interestingly, animals have lost the CYP710/CYP61 gene, presumably because it no longer fulfills any useful roles [53].

A comparison revealed striking sequence conservation between plant CYP710 proteins and fungal CYP61 proteins. The two substrate recognition site motifs, NX5GX2HX3RX6FTX3ALXY (position 86–114, SRS1) and FD/TFLFAA/SQDAS/TT/SS (position 268–280, SRS3) can be considered the signature of CYP61 or CYP710. For the motif in SRS3, D/T269, S/A274, and T/S279 can be used as phyla-specific residues (D269, A274, and T279 in plant CYP710s and T269, S274, and S279 in fungal CYP61s). Differences in the residues at these three positions might be related to the substrate preferences of CYP61 and CYP710. The residues D276 and A277 are specific and absolutely conserved in the CYP710 and CYP61 families of proteins. It can be inferred that these two residues are essential for their common configuration of their substrate-binding pockets. Details of the conserved residues will be useful for predicting specific substrates recognized by the enzymes from the CYP61 and CYP710 families.

In bryophytes, there have been few studies on sterol biosynthesis. Hatada et al. [54] analyzed the sterol profile in the bryophyte model plant *Marchantia polymorpha* L. The thalli contained typical phytosterols such as campesterol, β-sitosterol, and stigmasterol. A BLASTX analysis of the *M. polymorpha* genome against the *Arabidopsis thaliana* sterol biosynthetic genes confirmed the presence of all the enzymes responsible for sterol biosynthesis. The C22-sterol desaturase of bryophytes has not yet been sufficiently characterized. The genes encoding the C22-sterol desaturases have only been studied in the moss *P. patens* where two C22-sterol desaturases, CYP710A13 and CYP710A14, are responsible for the biosynthesis of stigmasterol, campesterol, and β-sitosterol [25]. It has been shown that *CYP710A13* and *CYP710A14* are expressed at all growth stages of *P. patens*, indicating the presence of C22-sterol desaturase activity during the entire life cycle. Both *CYP710A13* and *CYP710A14* contain single introns at +216 and +195 from the ATG, respectively. Interestingly, the CYP710A genes of *Arabidopsis* and rice lack introns. On the other hand, *CYP710B1* from the green alga *Chlamydomonas reinhardtii* contains a complex exon–intron structure that is not preserved in the *P. patens CYP710A* genes. The genes *CYP710A13* and *CYP710A14* encode polypeptides consisting of 503 and 502 amino acids with molecular masses of 57.410 and 57.028 Da, respectively. The predicted amino acid sequence of CYP710A13 is 81.2% identical to CYP710A14 and has 52.1% similarity to *Arabidopsis* CYP710A1 (At2g34500) and tomato CYP710A11 [35].

A comparison of the amino acid sequences of CYP710A proteins showed that the conserved sequence [F (L/M) FA (A/S) QDA (S/T) (S/T) S] in the substrate recognition site (SRS4) is present in higher plants and *P. patens*, but is absent in *C. reinhardtii* [25,35]. The conserved sequence is located precisely in the I-helix above the distal side of the heme group and is believed to be involved in the catalytic functions of P450 monooxygenases [55].

Recombinantly expressing the proteins CYP710A13 and CYP710A14 in a baculovirus/insect cell system showed that the enzymes exhibit a C22-sterol desaturase activity in the presence of β-sitosterol, resulting in the formation of stigmasterol [25]. The Km values for the metabolism of β-sitosterol by CYP710A13 (1.0 μM) and CYP710A14 (2.1 μM) are similar to those of higher plant CYP710A proteins. In *Arabidopsis* T87 cells overexpressing *CYP710A14*, the stigmasterol content was 20–72 times higher than in control T87 cells, further confirming the C22 desaturase activity of this enzyme. In *P. patens*, the presence of the end products of sterol biosynthesis confirms the existence of a complete biosynthetic pathway [25]. It seems clear that the pathway for sterol biosynthesis has been conserved during the evolutionary process of terrestrial plants. Elucidating the roles of plant sterols in plant development has been greatly facilitated by producing plants that lack C22 sterols using gene-knockout or RNA interference approaches [35,56].

In the Phytozome v12.1.6 database (https://phytozome.jgi.doe.gov/pz/portal.html (accessed on 15 May 2023), we found nucleotide sequences of CYP710A genes in three bryophyte species: *M. polymorpha*, *P. patens*, and *Sphagnum fallax* (Table 1). Analysis of these sequences showed that the exon–intron structure of the moss *CYP710A* genes varies from one to three exons. The structure of the *CYP710A* genes from these bryophytes corresponds to the structure of the annotated *P. patens CYP710A13* and *CYP710A14* genes described by Morikawa et al. [25]. As already mentioned, the *CYP710A* genes of higher plants lack introns, specifically those from *Arabidopsis*, tomatoes [35], and barley [56] that have been studied in detail. It remains unclear why introns have been lost during evolution, and why this simplification of the structure of the *CYP710A* genes has occurred. In the future, to further understand the evolution of the gene family, it will be important to study the gene structure, particularly the presence of introns and the number of exons.

To summarize, plant C22-sterol desaturases, by catalyzing the desaturation of the side chain of the sterol molecule, controls the ratio of *β*-sitosterol and stigmasterol, which is necessary for normal plant growth and development. Analyses of mutants with impaired stigmasterol biosynthesis confirmed that maintaining the normal balance of sterols is necessary for the optimal membrane state. The presence of the appropriate enzymes and synthesis of final products of sterol biosynthesis in *P. patens* confirms the existence of the whole pathway of sterol biosynthesis in mosses. Comparisons of the genomes of higher vascular and non-vascular model plants have provided strong evidence that sterol biosynthetic pathways have been conserved throughout the evolutionary radiation of land plants.

## 5. Stigmasterol Levels during Biotic and Abiotic Stresses

Stigmasterol has been reported to accumulate in various plant species in response to abiotic and biotic stress factors, for example, to unfavorable temperatures, drought, salt or UV treatment, and pathogen attack. Changes in stigmasterol content changes the ratio between sterols, which, in turn, can affect many metabolic processes. Here, we review the current information on the potential role of stigmasterol in the response of plants to stress.

### 5.1. Pathogen Infection

During evolution, plants have developed a variety of protective mechanisms to resist pathogens that include visible physical (morphological) and invisible structural and biochemical changes to their organs and tissues. Bacterial pathogens can colonize a host plant by growing between the cells and utilizing the nutrients in the apoplastic space. Therefore, it seems likely that there has been strong selection for strategies that reduce nutrient release into the apoplast; one mechanism is to reduce membrane permeability by manipulating sterol synthesis [47]. A study by Griebel and Zeier [8] showed that the inoculation of *Arabidopsis* with a virulent pathogen induces stigmasterol biosynthesis mediated by upregulation of *AtCYP710A1* gene activity. During the interaction of *Arabidopsis* plants with *Pseudomonas syringae* strains, stigmasterol is produced in the leaves between 10 and 48 h after inoculation and reaches levels of approximately 15 µg g^−1^ fresh mass. The amount of stigmasterol induced in the leaves by *P. syringae* is quantitatively similar to the increase in total salicylic acid, indicating that the C22 desaturation of *β*-sitosterol is a significant part of the metabolic response of *Arabidopsis* to a pathogen challenge. Furthermore, the conversion of *β*-sitosterol to stigmasterol is triggered after treatment of plants with elicitor molecules, such as flagellin 22 (flg22) and lipopolysaccharide (LPS), and the application of reactive oxygen species (ROS) [8]. The increased level of stigmasterol after H_2_O_2_ production by chloroplasts following contact with a pathogen may be an example of ROS signaling that increases plant defenses by influencing the properties of ordered membrane microdomains (rafts) [37]. In the leaves, the stigmasterol synthesized after pathogen assault is predominantly incorporated into plant membranes and might reduce their fluidity and permeability [8,57]. Paradoxically, according to Griebel and Zeier (2010) [8], enhanced stigmasterol formation after infection with the bacterial pathogen *P. syringae* increases the susceptibility of *A. thaliana* to bacterial attack. Interestingly, infection of cotton (*Gossypium hirsutum*) with different pathogens resulted in changes in stigmasterol concentrations in the opposite direction. For example, infection with the fungal pathogen *Verticillium dahliae* upregulated the expression of *GhCYP710A1*, increasing the ratio of stigmasterol to *β*-sitosterol [58], while infection with the nematode *Meloidogyne incognita* reduced the ratio of stigmasterol to *β*-sitosterol [59,60]. Therefore, it is clear that an increase in stigmasterol content is not a universal plant response to pathogens. However, critical analysis of the available data suggests that pathogen-induced changes in the ratio of stigmasterol/*β*-sitosterol can modulate plant defenses via the changes in the physicochemical characteristics of membranes (see Section 2). In addition, it seems likely that stigmasterol will influence downstream signaling by changing the number and/or properties of ordered membrane microdomains [61].

### 5.2. Salinization

Prolonged exposure of plants to salinity disrupts ionic homeostasis, which impairs physiological and biochemical processes, such as photosynthesis, protein synthesis, and lipid metabolism [62,63]. Free sterols are of great importance during salt stress because they can regulate the activities of membrane proteins and modulate membrane fluidity [64,65], which determine the effectiveness of membrane transport and homeostasis [66]. Most reports show that salinization increases the total content of sterols in plants [67], for example, in salt-adapted tomato calli [64], salt-tolerant wheat [68], and the halophyte *Kosteletzkya virginica* [69]. However, in addition to simply changing the total sterol content, salinization may also change the ratio between molecular species of sterols, which can influence the permeability of a membrane to salts due to the specific spatial structure of sterols. In particular, stigmasterol, campesterol, and cholesterol have a “planar” structure, while *β*-sitosterol is less planar [70]. It has been suggested that a shift to more planar sterols can decrease ion uptake [70,71]. In contrast, an increase in less-planar sterols can disrupt membrane packing, increasing the permeability of membranes to ions such as Cl^−^ [72]. For example, in salt-treated citrus, the exclusion of Cl^−^ is promoted by an increase in planar sterols such as cholesterol, stigmasterol, and brassica sterols [67,72]. In wheat caryopses, salt tolerance is promoted by high concentrations of the planar sterols campesterol and cholesterol [67]. Similarly, a decline in the level of *β*-sitosterol in salt-treated broccoli roots is associated with a reduction in the water permeability of the plasma membrane [73]. A study by Rosentsvet et al. showed that a characteristic feature of various halophytes, growing under different levels of salinity and soil moisture is the active synthesis of sterols with a C22 double bond, including stigmasterol [74]. Interestingly, exogenous applications of stigmasterol to germinating seeds of beans (*Vicia faba*) and flax (*Linum usitatissimum*) increase their tolerance to NaCl [75,76]. Therefore, the regulation of membrane permeability through changes in the sterol composition and ratio may play a significant role in the salt tolerance of plants.

The activation of appropriate signal transduction pathways in response to salinization may also be modulated by the interaction of stigmasterol with intracellular signaling molecules including Ca^2+^ and protons [7]. Plants grown under saline conditions have reduced root growth, but the addition of Ca^2+^ mitigates the harmful effects of salt stress [77]. One possible mechanism for this could be the stimulation of stigmasterol synthesis by Ca^2+^ [78], possibly by increasing *CYP710A* expression [7]. The Ca^2+^-induced increase in stigmasterol levels appears to activate plasma membrane H^+^-ATPase [79], leading to an increased extrusion of Na^+^ from cells [80]. Interestingly, stigmasterol and cholesterol can stimulate proton pumping, whereas *β*-sitosterol behaves as an inhibitor of pump activity [3]. Another role for stigmasterol in salt stress may be involvement in signaling by binding to the interactor synaptotagmin1 (ROSY1) protein, which modulates the response of plant cells to gravitational and salt stress [7,10]. Thus, although more studies are needed, stigmasterol may well be a participant in signaling events that occur in plant cells during the formation of a stress response to abiotic factors, in particular, salinization.

### 5.3. Unfavorable Temperatures

In nature, plants often experience low or high temperature stresses. Plants experience greater temperature fluctuations than animals, which can regulate their body temperature or change their location to avoid unfavorable temperatures. During evolution, plants developed their own ways of acclimating to temperature fluctuations, and it has been suggested that this may occur through the regulation of the amounts of ethylsterols such as *β*-sitosterol and stigmasterol. As discussed above, unlike cholesterol, ethylsterols have additional ethyl groups branched at C24. These sterols can interact with glucosyl cerebrosides and thereby increase membrane adhesion due to the formation of membrane domains, thus facilitating adaptation to large temperature fluctuations [17]. Other potential way that phytosterols could facilitate the acclimation of plants to temperature is through regulating membrane fluidity and permeability [57], and the ability of sterols to resist the oxidation caused by ROS [81]. Several reports have demonstrated that heat stress increases the total sterol content of plants. For example, exposure to high temperatures both in a greenhouse (20/10 °C, 27/17 °C, 35/25 °C day/night) and in a field increased the total levels of sterols in soybean seeds [82]. Interestingly, in sunflower seeds, heat stress increased the sterol content by increasing *β*-sitosterol and campesterol, while the level of stigmasterol remained unchanged [83]. It appears that the effects of temperature stress on the sterol content are highly organ-specific. Previously, we showed that in wheat seedlings, low positive temperatures increase the relative content of the methylsterol campesterol in the roots, while in contrast, in the leaves, the contents of the ethylsterols *β*-sitosterol and stigmasterol were increased. Furthermore, the level of C22-sterol desaturase transcripts was much higher in the roots exposed to cold than in the leaves, confirming the organ-specificity of the effects of temperature on sterol levels [49]. Despite the lack of changes in the stigmasterol content in some plant parts following heat stress, an analysis of the effects of temperature stress on membrane leakage, chlorophyll content, growth, and viability in the *Atcyp710a1* mutant and *AtCYP710A1*-overexpressing lines showed that the *AtCYP710A1* gene is involved in conferring low and high temperature tolerance in *Arabidopsis* [45].

Thus, the literature and our own recent unpublished results demonstrate the involvement of stigmasterol in the response of plants to unfavorable temperatures, especially low temperature, by increasing membrane fluidity due to the presence of an ethyl group and a C22 double bond in the structure of this phytosterol.

### 5.4. Drought

Drought is becoming an increasingly serious problem for plants, particularly crops, due to global climate change. In *Arabidopsis*, Pose et al. [84] identified a drought-hypersensitive/squalene epoxidase 1–5 (dry2/sqe1–5) mutant that has a defective squalene epoxidase1 (SQE1) gene. The mutant displayed a variety of properties that make it sensitive to drought, which suggested that dry2/sqe1–5 regulates stomatal closure, proline accumulation, chlorophyll degradation, and the induction of stress-related genes. Furthermore, the mutant exhibited pleiotropic developmental defects that were correlated with various changes in the sterol composition of the shoot and the root. It was concluded that a reduction in SQE1 activity changes the composition of sterols, which in turn leads to impaired functioning of NAD(P)H oxidase and ROS production, which are probably responsible for the developmental defects observed in the mutant [84]. Along with other detrimental consequences, drought damages cell membranes by breaking down membrane lipids. Di-4-ANEPPDHQ fluorescence and spectral phasor analyses showed that the drought-sensitive dry2/sqe1–5 mutant displays higher membrane fluidity than the wild type [85]. Therefore, these sterols probably play important roles in drought tolerance by strengthening cell membranes.

The roles of sterols in response to drought stress were investigated in the seedlings of two rice varieties (*Oryza sativa*), cultivar N22 (drought tolerant) and cultivar IR64 (drought susceptible) [86,87]. As the seedlings matured, the concentrations of campesterol, stigmasterol, and *β*-sitosterol increased. The drought-tolerant cultivar showed a higher content of sterols and their corresponding sterol esters, and during drought stress, this content doubled. In particular, it was found that in the drought-tolerant N22 rice seedlings, with increasing dehydration stress, the level of *β*-sitosterol and campesterol and their esters, and the activity of 3-hydroxy-3-methyl-glutaryl-CoA reductase (HMGR), the key enzyme of the mevalonate pathway, were elevated. These results clearly suggest that the greater drought tolerance of N22 is a consequence of its ability to accumulate sterols and their esters in comparison with the drought-sensitive cultivar [86]. This is of particular interest given that the moss is strongly poikilohydric, and suggests that sterols may be important generally in desiccation tolerance.

### 5.5. UV Radiation

Ultraviolet (UV) radiation is detrimental to the most living organisms. Like other stress factors, exposure to UV-B radiation, which includes electromagnetic radiation from 280 to 315 nm, markedly elevates the cellular levels of ROS and can cause oxidative damage to plants [88]. Plants respond to this stress by inducing a complex antioxidant defense system involving various enzymes and specialized metabolites, and also by accumulating various compounds that absorb excessive UV radiation [89,90]. While little information is available so far, several studies have shown that sterols may contribute to the response of plants to UV stress. Compounds derived from the cholesterol and isoprenoid biosynthetic pathways have been shown to be a part of the UV-B response in grapevine leaves [91]. Specifically, exposing grapevine vegetative tissues to low levels of UV-B increases levels of membrane-bound *β*-sitosterol, stigmasterol, and lupeol, which, as discussed above, can improve membrane stability [91]. Treating leaves of the Indian medicinal plant *Withania somnifera* with UV increased the levels of stigmasterol (ethylsterol), while the levels of methyl sterols, campesterol, crinosterol, and cholesterol decreased significantly. Thus, the proportion of more hydrophobic ethylsterols in membranes increased, which can influence membrane stability. In the roots of *W. somnifera*, UV irradiation decreased the levels of cholesterol while two new sterol esters, stigmasterol acetate and *β*-sitosterol oleate, were synthesized [5]. Apart from general effects on membrane stability, some sterols, e.g., *β*-sitosterol, may contribute to the total antioxidant activity [92]. Ahmed and Schenk [93] demonstrated that UV increased the concentrations of fungisterol, chondrillasterol, and dihydrochondrillasterol in the microalgae *Pavlova lutheri*, and it seems likely that these compounds can play an important role in the repair of the damaged cellular membranes. Thus, many results demonstrate that UV exposure significantly affects sterol metabolism, and the shifts in the ratio of sterols can improve membrane stability and reduce the harmful effects of UV-B damage.

### 5.6. Heavy Metals

The term “heavy metals” technically only includes elements with a specific gravity greater than five, but biologists often use this term to refer to a wide range of metals and metalloids that are toxic to plants, such as copper, iron, manganese, zinc, nickel, cobalt, cadmium, and arsenic. Metals and metalloids, although normal constituents of soils, sometimes occur at excessive concentrations and can then be harmful to plants. In general, the treatment of plants with heavy metals reduces the sterol content of the roots and increase the permeability of the root plasma membrane. For example, the application of 50 μM Cu to plant roots increases K^+^ leakage and decreases the total lipid content of the plasma membrane, specifically leading to a higher phospholipid content and a lower content of free sterols, steryl glycosides, and acylated steryl glycosides [94]. The role of sterols in the resistance of plants to heavy metal toxicity has not been studied in detail, but information exists showing that the steroidal plant hormones from the family of brassinosteroids, which are campesterol derivatives, can help plants to remove toxic metals [95]. Brassinosteroids reduce the uptake of toxic metals by altering cell permeability and alleviating damage by activating defensive enzymes [96]. Detailed studies of the role of sterols in the aluminum resistance of plants carried out by Wagatsuma et al. [20,97] demonstrated that changes in the lipid composition of the plasma membrane can reduce the penetration of aluminum and thus increase the resistance of plants to aluminum. As discussed above in Section 5.2, due to their different chemical structures, sterols differ in their effects on membrane permeability. Specifically, for stigmasterol, the presence of trans-oriented unsaturation at the C22 of the stigmasterol molecule can increase membrane permeability. Therefore, decreasing the content of stigmasterol can lower plasma membrane permeability, thereby increasing resistance to aluminum. Thus, the stigmasterol content in cell membranes can serve as an important factor regulating membrane permeability and protecting cells from heavy metal toxicity.

### 5.7. Stress Phytohormones

One experimental approach to test the effects of stresses on plants under laboratory conditions is to treat plants with exogenous “stress” phytohormones, e.g., abscisic acid (ABA), methyl jasmonate (MeJA), and salicylic acid (SA). ABA controls many vital processes in plants, such as regulation of seed and bud dormancy, early stages of ontogenesis, stomata closure, and plant responses to stress factors, such as drought, salinity, and low temperature [98]. Plant sterols have been shown to prevent ABA-induced disruption of artificial phosphatidylcholine/phosphatidylethanolamine bilayers. The effect of ABA on membranes is equally inhibited by plant sterols and cholesterol, which may indicate the participation of plant sterols in the control of the negative effect of ABA on membranes [50,99]. MeJA and SA are involved in the signal transduction pathways that increase the resistance of plants to pathogen attack, e.g., by increasing in the concentration of metabolites participating in defense mechanisms [100]. He et al. [101] suggested that sterols may work as regulatory molecules in concert with hormone-mediated signaling pathways in the control of plant metabolism and development. This control can be achieved by maintaining the ratio of 24-methyl/ethylsterols, which is important for ensuring the functional activity of membranes under stress conditions. In our work, significant changes in the ratio of 24-methyl/ethylsterols towards an increase in the proportion of methylsterols were observed in the wheat roots under the action of MeJA and in the wheat leaves under the action of ABA. The content of stigmasterol in the roots and leaves increased under the action of ABA and SA [50]. The ratio of *β*-sitosterol and stigmasterol, in turn, is determined by the activity of C22-sterol desaturase. Our studies have shown that phytohormone-induced changes in the activity of the C22-sterol desaturase of wheat seedlings are organ-specific. In particular, C22-sterol desaturase in the roots was more sensitive to ABA treatment, in contrast to the leaves of wheat seedlings. On the contrary, C22-sterol desaturase in leaves, but not in roots, was susceptible to the action of SA [50]. Thus, our data indicate the presence of hormone-mediated regulation of plant sterol composition and the possible involvement of stigmasterol in plant cell hormonal signaling.

### 5.8. Summary of the Role of Stigmasterol in the Response of Plants to Stresses

Table 2 summarizes the multiple studies that have shown the influences of stress on the levels of stigmasterol in plants. Many stressors, such as pathogens, UV radiation, salinity, low temperature, and the stress phytohormones ABA and SA elevate the levels of stigmasterol, all suggesting that stigmasterol is a stress sterol. In contrast, high temperature appears to have little effect on the levels of stigmasterol in plant cells, while heavy metal treatment reduced the stigmasterol content. Thus, it is clear that changes in the stigmasterol level in plant cells are stress-specific. However, to understand the precise roles of stigmasterol in the responses of plants to stress will require further study.

## 6. Application of Stigmasterol in Medicine

As mentioned in the Introduction, mammals can only synthesize cholesterol and its derivates and therefore they can only obtain phytosterols supply from food. There are currently ca. 250 plant sterols that differ in biological activities and accessibility, but at present, stigmasterol is attracting increased attention due to its diverse pharmacological properties [102]. Stigmasterol is naturally rich in common foods such as banana, cabbage, peanut, soybean, sunflower, corn [103], and also seaweed [104]. Stigmasterol has been isolated from various medicinal plants such as *Butea monosperma* (Lam.) Taub. (Fabaceae), *Edgeworthia gardneri* (Wall.) Meisn. (Thymelaeaceae), *Parkia speciosa* Hassk. (Fabaceae), and *Calotropis gigantea* (L.) Dryand. (Apocynaceae) [105]. The medicinal effects of stigmasterol have been widely reviewed in the literature [106,107]. In vitro and in vivo studies demonstrated the anti-osteoarthritis [108], anti-inflammatory [109], anti-diabetic [107,110], immunomodulatory, antiparasitic, antifungal, antibacterial, antioxidant [111], and neuroprotective properties of stigmasterol [106]. Moreover, various biological and pharmaceutical properties of stigmasterol have been shown such as analgesic properties [112], maintaining psychiatric status [113], lowering blood cholesterol levels [114], improving learning and memory abilities [115], and protecting against *Leishmania* [116]. In particular, stigmasterol can perform neuroprotective functions in disorders of the central nervous system, such as Alzheimer’s disease, multiple sclerosis, and amyotrophic lateral sclerosis/parkinsonism dementia [117]. It was found that stigmasterol decreases the cerebral activity of amyloidogenic enzymes in mice [118]. Unlike cholesterol, phytosterols, especially stigmasterol, can cross the blood–brain barrier and accumulate in the brain [119]. It has been shown that stigmasterol can significantly reduce the level of amyloid *β* in human neuroblastoma cells. Furthermore, stigmasterol exerts neuromodulatory effects [115] by activating glutamatergic neurotransmission and inhibiting acetylcholinesterase activity [120,121]. Moreover, recently, stigmasterol was reported to have anti-tumor potential either in vivo or in vitro against several types of cancer, for example, lung cancer [122], gallbladder cancer [123], gastric cancer [124], and ovarian cancer [107,125]. Although displaying a great diversity of positive medicinal effects, stigmasterol demonstrates low cytotoxicity [126]. The mechanisms of the anti-cancer effects of stigmasterol are based, among other properties, on its ability to increase ROS production and Ca^2+^ levels in the cytosol and mitochondria, cause mitochondrial depolarization, induce apoptosis, suppress cell migration, and inhibit the activity of angiogenesis genes in tumor cells [106]. Interestingly, according to some data in the literature, the neuroprotective effects of stigmasterol are caused by its ability to reduce oxidative stress by decreasing the levels of ROS and lipid peroxidation, and its ability to inhibit apoptosis [127,128]. However, the molecular mechanisms underlying the neuroprotection by stigmasterol are currently not fully understood. A study by Haque et al. [129] showed that stigmasterol induces mitophagy in H/R (hypoxia/reoxygenation)-exposed hippocampal cells. It is known that the activation of autophagy plays an important neuroprotective role after hypoxic injury [130]. Transcriptome studies have demonstrated that stigmasterol can function as a liver X receptor (LXR) agonist and protect neurons from pathological excitation by modulating N-methyl-D-aspartate (NMDA) signaling and promoting mitophagy [131]. In silico studies of the interaction between stigmasterol and LXR *β* have shown that stigmasterol forms multiple hydrogen bonds with the GLU281 and ARG319 LXR *β* residues in an orientation similar to that of other endogenous steroid–nuclear receptor complexes [132]. Thus, stigmasterol is a unique plant sterol that exhibits diverse pharmacological activities (Table 3), and further study seems likely to increase the prospects for its use in medicine.

## 7. Conclusion: Stigmasterol as a Stress Sterol of Plants

**Figure 2:**
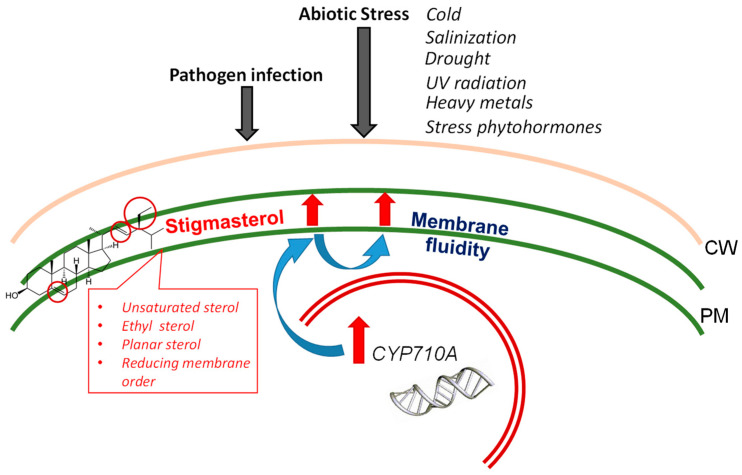
Stigmasterol involvement in stress response of cells. Stigmasterol is an unsaturated ethylsterol, which can reduce membrane order. The biosynthesis and accumulation of stigmasterol in response to abiotic and biotic stresses result in an increase in membrane fluidity and promote the adaptation of plants to environmental changes.

The unsaturated sterol stigmasterol is the end product of the stigmasterol branch of the mevalonate biosynthetic pathway in plants. The current research is focused on understanding the physiological meaning of variations in stigmasterol production in plants. At present, experiments on the involvement of stigmasterol in normal plant development and in stress responses of plants have given results that are unclear and even contradictory. While the absence of stigmasterol production does not always lead to the loss of plant viability, marked changes in stigmasterol content in response to various stresses may indicate that this sterol is part of a “general plant stress response”. Interestingly, extremophilic plants tend to have much higher stigmasterol contents than stress-sensitive plants. As emphasized in this review, changes in the stigmasterol content of plants are strongly dependent on the organs of the plant and stage of development, and any specific stress the plant is experiencing. Structural features of the stigmasterol molecule, e.g., the presence of an additional double bond and a generally planar 3-D shape, help to maintain the fluidity and permeability of plant membranes under stress conditions (Figure 2). However, the present state of the literature leaves us with more questions than answers. Further research is necessary to unravel the detailed physico-chemical mechanisms of stigmasterol’s involvement in plant life and its possible application in plant breeding and medicine.

## Figures and Tables

**Table 1:** Structure of *CYP710A* genes in different species of bryophytes.

Parameter	*Marchantia polymorpha* (Mapoly0103s0038)	*Physcomitrium patens (Pp3c2_27810V3)*	*Physcomitrium patens (Pp3c1_11690V3)*	*Sphagnum fallax (Sphfalx0121s0046)*
Genomic seq. (bp)	4049	3340	3177	3517
Transcript seq. (bp)	3791	2853	2524	2619
CDS seq. (bp)	1518	1512	1509	1578
Peptide seq. (aa)	505	503	502	525
Exon number	1	2	2	3

**Table 2:** The effects of stress on stigmasterol metabolism in plants and the effects of exogenous applications of stigmasterol on stress tolerance.

Stress	Stigmasterol Stress Response	References
Pathogen infection	Inoculation of *Arabidopsis* with *Pseudomonas syringae* induces stigmasterol biosynthesis.	[8]
	The level of stigmasterol increases after H_2_O_2_ production by chloroplasts following contact with a pathogen.	[37]
	Infection with the fungal pathogen *Verticillium dahliae* upregulates the expression of *GhCYP710A1*, increasing the ratio of stigmasterol to *β*-sitosterol.	[58]
	Infection with the nematode *Meloidogyne incognita* reduces the ratio of stigmasterol to *β*-sitosterol.	[59,60]
Salinization	Salt treatment of citrus leads to both the exclusion of Cl^−^ and an increase in stigmasterol.	[67,72]
	Halophytes growing under different levels of salinity and soil moisture actively synthesize stigmasterol.	[74]
	Exogenous applications of stigmasterol to germinating seeds of *Vicia faba* and *Linum usitatissimum* increase their tolerance to NaCl.	[75,76]
Unfavorable temperatures	In sunflower seeds, heat stress increases the content of *β*-sitosterol and campesterol, while the level of stigmasterol remains unchanged.	[83]
Drought	In the seedlings of two varieties of *Oryza sativa* during drought stress, the tolerant cultivar accumulated more stigmasterol than the sensitive cultivar.	[86,87]
UV radiation	Treating leaves of the medicinal plant *Withania somnifera* with UV increases the level of stigmasterol.	[5]
Heavy metals	A decrease in stigmasterol can lower plasma membrane permeability, thereby increasing resistance to aluminum.	[20,97]
Stress phytohormones	The content of stigmasterol in the roots and leaves of *Triticum aestivum* increases following treatment with ABA and SA.	[50]

**Table 3:** The potential medical applications of stigmasterol.

Medicinal Effect of Stigmasterol	Animal Model System(s)	Possible Mechanisms	Reference(s)
Anti-osteoarthritis	Rabbit	Stigmasterol could be related to six targets, namely, NCOA2, PGR, PTGS1, PTGS2, RXRA, and NR3C2. These primary target genes are linked to signaling pathways involved in cartilage degeneration in knee OA, including the PI3K–Akt signaling pathway and the TNF-α signaling pathway.	[108]
Anti-inflammatory	Mice and the BV2 cells	Stigmasterol therapy markedly inhibited the expression of pro-inflammatory mediators, including iNOS, IL-6, IL-1β, COX-2, and TNF-α, and upregulated the expression of anti-inflammatory cytokines such as IL-10 via negative regulation of p38MAPK expression and NF-kBp65 (suppression of p-IKB-α activation) in the joints.	[109]
Anti-diabetic	L6 cells	Stigmasterol binds to sirtuin 4, an NAD-dependent deacylase enzyme that down-regulates leucine and glutamate-dehydrogenase-induced insulin secretion.	[107,110]
Neuroprotective	Hippocampal neuronal cells, hippocampal neurons, cultures of brain neurons	The neuroprotective effects of stigmasterol are caused by its ability to reduce oxidative stress by decreasing the levels of ROS and lipid peroxidation and its ability to inhibit apoptosis. A study by Haque et al. showed that stigmasterol induces mitophagy in H/R (hypoxia/reoxygenation)-exposed hippocampal cells. It is known that the activation of autophagy plays an important neuroprotective role after hypoxic injury. Transcriptome studies have demonstrated that stigmasterol can function as a liver X receptor (LXR) agonist and protect neurons from pathological excitation by modulating N-methyl-D-aspartate (NMDA) signaling and promoting mitophagy. In silico studies of the interaction between stigmasterol and LXR *β* have shown that stigmasterol forms multiple hydrogen bonds with the GLU281 and ARG319 LXR *β* residues in an orientation similar to that of other endogenous steroid–nuclear receptor complexes.	[106,127,128,129,130,131,132]
Anti-tumor potential			
Lung cancer	NCI-H1975 cells	Proliferation	[107,122]
Gall bladder cancer	Human gall bladder cancer cells	Apoptosis	[107,123]
Gastric cancer	Blood cells	Proliferation, apoptosis, autophagy	[107,124]
Ovarian cancer	ES2 and OV90 cells	Apoptosis, migration (ROS, calcium)	[107,125]

## Data Availability

Not applicable.
